# Organic Fertilization Assembles Fungal Communities of Wheat Rhizosphere Soil and Suppresses the Population Growth of *Heterodera avenae* in the Field

**DOI:** 10.3389/fpls.2020.01225

**Published:** 2020-08-07

**Authors:** Wei Qiu, Huiqing Su, Lingyun Yan, Kaiyan Ji, Qian Liu, Heng Jian

**Affiliations:** Department of Plant Pathology and MOA Key Laboratory of Pest Monitoring and Green Management, China Agricultural University, Beijing, China

**Keywords:** *Heterodera avenae*, organic fertilizer, microbiome assembly, rhizosphere, soil fungi communities, suppression, population growth, wheat field

## Abstract

*Heterodera avenae* (cereal cyst nematode, CCN) infects wheat and other cereal crops and causes severe losses in their yield. Research has shown that CCN infestations can be mitigated by organic fertilization in wheat fields, but the mechanisms underlying this phenomenon are still largely unknown. In this study, the relationships among CCN, soil properties, and soil fungal communities with organic fertilizer (OF) or chemical fertilizer (CF) and without fertilizer (CK), were investigated for two years in a wheat field in Henan province, China. Our results showed that the concentrations of soil total N, total P, available P, available K, and organic matter were all promoted by the OF treatment at the jointing stage of wheat, coinciding with the peak in egg hatching and penetration of wheat root by CCNs. Soil total N correlated positively (R^2^ = 0.759, *p* < 0.05) with wheat yields but negatively (R^2^ = 0.663, *p* < 0.01) with Pf/Pi (index of cyst nematode reproduction), implying the regulated soil property contributes to suppressing CCN in the OF treatment. Furthermore, fungal community α-diversity (Shannon and Simpson) and β-diversity (PCoA) of rhizosphere soil was improved under the organic fertilizer treatment. The fungal genera negatively associated with the Pf/Pi of CCN were highly enriched, which included *Mortierella* and *Chaetomium*, two taxa already reported as being nematophagous fungi in many other studies. These two genera were heavily surrounded by much more related fungal genera in the constructs co-occurrence network. These results suggested that the OF treatment shifted soil fungal community functioning towards the suppression of CCN. Taken together, the suppressed cyst nematode reproduction with the assembly of fungal communities in the rhizosphere led to greater wheat yields under organic fertilization. These findings provide an in-depth understanding of the benefits provided by organic fertilization for developing sustainable agriculture.

## Introduction

*Heterodera avenae* (cereal cyst nematode, CCN) is among the most important soil-borne pathogens infesting cereal crops worldwide. The wheat yield losses caused by *H. avenae* can range from 10% up to 100% in some infected fields (Bonfil et al., 2004; Smiley et al., 2017; Yang et al., 2019). In modern intensive agriculture, usually a synthetic nematicide is used to manage this nematode and safeguard wheat yields (Zhang et al., 2016), and while its control efficacy is significant, such nematicides are expensive and they also pollute the local environment (Peng et al., 2009), which over the long-term is unsustainable (Bridge, 1996). Furthermore, intensive agriculture systems based on the continuous use of mineral fertilizers have led to reductions in soil biodiversity and aggravated the infestation of CCN (Hu et al., 2014; Matute et al., 2018). Thus, taking an environmentally friendly view of fertilization in crop management, which could reduce both soil pollution and the damages from CCN, is urgently needed for developing truly sustainable agroecosystems.

Research has revealed that the diversity and density of soil nematodes can change depending on the fertilization type used in fields (Liang et al., 2009; Diacono and Montemurro, 2010; Al-Hazimi and Dacbah, 2014; Hu et al., 2014). Nitrogen fertilization significantly enhances the contents of soil ammonium and nitrate nitrogen, which have the potential to negatively impact soil nematodes (Zhao et al., 2014; Song et al., 2015). Similarly, negative effects upon soil nematodes were observed when a high level of phosphate fertilizer was applied in forest soils (Zhao et al., 2014). Specifically, adding urea or calcium superphosphate alone can significantly inhibit the abundance of CCN (Yang et al., 2008; Al-Hazimi and Dacbah, 2014). Ammonium sulfate fertilizer was found to significantly limit the abundance of soil nematode communities (Ikoyi et al., 2020), whereas potassium sulfate promoted the occurrence of CCN-associated disease in wheat plants (Yang et al., 2008). Different fertilization types may change the main food sources of soil nematodes, thereby changing the community structure of soil nematodes (Bjørnlund et al., 2006; Zhao et al., 2014). In particular, organic inputs to soil tend to change the proportion of plant parasitic nematodes most significantly (Liu et al., 2016), and some studies have demonstrated that not only can organic fertilizers fertilize the soil and increase wheat yields but also effectively reduce the damage to the crops caused by CCN (Diacono and Montemurro, 2010; Hu et al., 2014). However, such research on how different fertilization treatments affect soil nematodes are usually limited to investigating the changed chemical properties of treated soil. The key factors, abiotic and biotic, driving CCN infestations under different modes of fertilization are still largely unknown.

A large abundance of nematode antagonistic fungal consortia will accumulate on plant parasitic nematode suppressive soil (Raaijmakers and Mazzola, 2016; Hamid et al., 2017; Botelho et al., 2019; Topalovic et al., 2020). For instance, the fungi *Cladosporium* and *Syncephalastrum* were significantly enriched in suppressive coffee farm soils infested with the root-knot nematode *Meloidogyne exigua* (Botelho et al., 2019). Both *Purpureocillium* and *Pochonia* were more abundant in soybean cyst nematode suppressive soils under long-term monoculture conditions (Hamid et al., 2017). Other taxa of nematode antagonistic fungi, such as *Dactylella*, *Nematophthora*, *Trichoderma*, *Hirsutella*, *Haptocillium*, *Catenaria*, *Arthrobotrys*, *Dactylellina*, *Drechslerella*, *Chaetomium*, and *Mortierella* were also detected in suppressive soils (Kerry and Crump, 1980; Ashrafi et al., 2017; Topalovic et al., 2020). Apart from those specific fungi, there is evidence that communities of fungal consortia can inhibit the growth and activity of soil-borne pathogens in disease suppressive soils (Weller et al., 2002; Dudenhöffer et al., 2016). Additionally, substrate-mediated shifts in soil microbial communities were found associated with the transition of *Verticillium* wilt-conducive soils to -suppressive soils, suggesting that soil-borne pathogen suppressive soils can arise *via* manual amendment (Mueller and Sachs, 2015; Inderbitzin et al., 2018). Recently, nitrate fertilization improved the resistance of cucumber to *Fusarium* wilt disease alongside the assembly of fungal communities in the plant’s rhizosphere (Gu et al., 2020), pointing to a underappreciated factor by which fertilization engenders disease suppressive soils. Although belowground associations between plant parasitic nematodes and soil fungal communities have been widely studied, the mechanisms underpinning fertilization-driven shifts in fungal community functioning towards suppression of the CCN pathogen are less known.

Here, we investigated the different effects of organic versus inorganic fertilizers on (i) the population growth of cereal cyst nematode (CCN) in wheat plants, and (ii) the soil fungal communities in a wheat field. Based on our results, we then attempt to clarify the relationships between CCN and soil fungal communities under the application of organic fertilizers. Finally, we comment on feasible technologies and approaches to alleviate CCN damage to staple crops to increase their productivity in sustainable agricultural systems.

## Materials and Methods

### Experiment Design and Soil Sampling

The experimental site was located at the Kai-Feng Experimental Station of China Agricultural University in Henan Province, North China Plain (34°76′ N and 114°27′ E), where there is a high incidence of *Heterodera avenae* disease (Yuan et al., 2014). The mean annual temperature is 16.5°C, ranging from a minimum of 2°C in January to a maximum of 29.5°C in July, and mean annual precipitation is approximately 621 mm, of which 60% occurs from July to September.

The fertilization experiment was designed to test three treatments: *organic fertilizer* (OF: applied at 15 t ha^-1^, according to the report by Hu et al. (2014), soil plant-parasites nematodes could be suppressed under this dosage; mostly chicken manure, with a mean nutrient content of N = 18.63 g kg^-1^, total P = 15.67 g kg^-1^, total K = 18.51 g kg^-1^, and organic matter = 363.56 g kg^-1^), *chemical fertilizer* (CF: 234 kg ha^-1^ of N, 72 kg ha^-1^ of P_2_O_5_, 99 kg ha^-1^ of K_2_O; local traditional fertilization), and a *control group* (CK: no fertilization). Each treatment had three replications. Each replication consisted of a plot 8 m × 8 m in size (L × W), with all 9 plots laid out in a completely randomized arrangement. All plots were planted with wheat (*Triticum aestivum* cv. ‘Zhoumai 22’) in winter (from October to June) and with maize (*Zea mays* L.) in summer (from June to October) on an annual rotation, which is the typical cropping system used in this region. For the OF treatment, organic fertilizer was applied on the day before wheat sowing. As to the CF treatment, which is the conventional mode of fertilization used by farmers in this region, 2/3 of the total fertilizer was applied prior to wheat sowing and the other 1/3 applied at the end of February. Except for the different fertilizers, all field management practices were the same, and no fertilizer was applied during the planting of maize. The fertilization experiment was conducted from October in 2017 to June in 2019.

For soil fungal community analysis, rhizosphere soil samples were collected at seven time-points: before sowing, and the seedling, wintering, regreening, jointing, heading, and harvest stages of wheat. To obtain the rhizosphere soils, excess soil was first removed by manually shaking the roots of wheat, leaving an approximately 2-mm layer of soils still attached to the roots. Then, using a sterilized brush, the root-attached soil material was collected as a rhizosphere soil sample (Edwards et al., 2015). This material from nine random sampling points [each sampling point contains an area 15 cm × 15 cm × 20 cm in size (L × W × D)] in each plot was then pooled into one sample (approximate 60 g soils); hence there were three replicated samples per fertilizer treatment. In addition, from each sample, 20 g of rhizosphere soils were immediately frozen in liquid nitrogen for the fungal community analysis, and the remainder (approximate 40 g soils) used for the analysis of their chemical properties. The wheat grain weight in an area 1 m × 2 m in size (L × W) per plot was measured as crop yield and recorded.

### CCN Cyst Investigation and Chemical Properties’ Analysis

To investigate number of cysts of CCN, bulk soil samples before wheat crop’s sowing and after its harvest were collected. There were three soil samples were collected for per plot, each soil sample contained three randomly sampling sites, each site was sampled in an area 15 cm × 15 cm × 20 cm (L × W × D). There were 27 soil samples (3 samples × 9 plots = 27) were collected. 500 g soil from each soil sample was used to extract the cysts by following the methodology of Krusberg et al. (1994). Next, the number of recovered cysts per sample was counted under a stereoscope (Olympus, Japan). An index Pf/Pi of cereal cyst nematodes was calculated by cyst numbers at the harvesting of wheat comparing to that before sowing.

Soil’s pH and nutrient concentrations (organic matter, available P, total P, available K, total K, and total N) were measured according to the *Soil Physicochemical Analysis Handbook* (Bao, 2000), using air-dried rhizosphere soil samples. Briefly, a 10 g soil sample was placed in a 200 ml flask with 50 ml of distilled water and shaken for 3 min and then filtered. A 20 ml aliquot from the filtrate was used to determine the pH value with a pH meter (METTLER TOLEDO, Switzerland). The organic matter was digested from 0.2 g soils with 0.4 mol L^-1^ K_2_Cr_2_O_7_-H_2_SO_4_ applying additional heat (170°C) in oil for 5 min and determined by titrimetric analysis. The available P was extracted from 2.5 g soils with 0.5 mol L^-1^ NaHCO_3_ for 30 min and total P was extracted from 1.0 g soils with HClO_4_-H_2_SO_4_ digested for 1 h, then the digestive solutions were analyzed using a spectrophotometer (LASPEC, China). The available K was extracted from 2.5 g soils with 1.0 mol L^-1^ NH_4_OAc for 30 min and total K was extracted from 0.2 g soils with 2.0 g solid granular NaOH applying additional heat (720°C) for 15 min, then the digestive solutions were analyzed using a flame photometer (Alpha Chemika, England). Total N was determined with 1.0 g soils by a semi-micro Kjeldahl digestion followed by ammonium distillation and titrimetric determinations.

### Fungal Diversity and Community Analysis

To extract the total DNA from each rhizosphere soil sample (0.5 g), the FastDNA^®^ Spin Kit for Soil (MP Biomedicals, USA) was used by following the manufacturer’s instructions. The purity and concentration of all extracted DNA were both quantified by a NanoDrop™ spectrophotometer (Thermo Fisher Scientific, USA). The ITS1-ITS2 hypervariable region of fungi was amplified using the specific primers ITS1F (5’-CTTGGTCATTTAGAGGAAGTAA-3’) and ITS2R (5’-GCTGCGTTCTTCATCGATGC-3’) (Adams et al., 2013). In brief, the PCR system consisted of 4 μl of 5×FastPfu Buffer, 2 μl of 2.5 mM dNTPs, 0.8 μl of each primer (5 μM), 0.4 μl of FastPfu polymerase (TIANGEN, China), 0.2 μl of BSA, and 10 ng of template DNA, which was amplified in a total volume of 20 μl. The thermal-cycling conditions used: denaturation at 95°C for 3 min, then 35 cycles at 95°C for 30 s, annealing at 55°C for 30 s, with an extension at 72°C for 45 s, followed by 10 min at 72°C. The PCR products were purified using a PCR Clean-UpTM kit (MO BioLabs, USA) and then sent to the Majorbio Company (Shanghai, China) for sequencing using the Illumina MiSeq PE300.

### Bioinformatics Analysis

Barcodes and primers were removed after quality control, any low-quality sequences were filtered out using Trimmomatic (v0.33, http://www.usadellab.org/cms/?page=trimmomatic) and the remainder merged using the Flash tool (v1.2.11, https://ccb.jhu.edu/software/FLASH/). Those merged sequences having > 97% similarity were assigned to the same operational taxonomic unit (OTU), using Uparse software (v7.0.1090, http://www.drive5.com/uparse/) (Edgar et al., 2011). The OTUs’ abundance levels were normalized based on the sample having the least number of sequences, which was 31,639. To ensure robust comparisons among the samples, all subsequent analyses were made using this normalized data set. Taxonomic characterization of the representative sequences of fungal OTUs was performed using the Unite database (v8.0, https://unite.ut.ee/), by applying the BLAST method with a 0.7 similarity threshold (https://blast.ncbi.nlm.nih.gov/Blast.cgi). The richness estimators (Sobs and Chao1), diversity indices (Shannon and Simpson) and coverage indices (Coverage) were determined using Mothur software (v1.30.1 http://www.mothur.org/). The FUNGuild approach was used for making fungal community predictions (Nguyen et al., 2016).

### Statistical Analysis

To test for differences in fungal α-diversity (Sobs, Chao1, Shannon, Simpson, or Coverage) among the three treatments, the Student’s t test was used. In the β-diversity analyses, principal coordinate analyses (PCoA) utilizing the bray-curtis distances were used to evaluate the differences among treatments. Meanwhile, redundancy analysis (RDA) was relied upon to examine the relationships among a subset of selected soil environmental properties and the soil fungal communities. PCoA, analysis of similarities (ANOSIM), and RDA were all carried out using the “vegan” package in R (v.3.5.1; https://cran.r-project.org). Linear discriminant analysis (LDA) coupled with effect size determination (LEfSe) was conducted to identify significantly different fungal taxa among the OF, CF, and CK treatments. The LDA score threshold was set to 2.5, and this analysis done online to obtain the LEfSe (http://huttenhower.sph.harvard.edu/galaxy/).

To construct the co-occurrence network of fungal genera in each treatment, only the abundant genera were used (the 50 most abundant). Because the data did not conform to the assumptions of the general linear model, significant correlations between any two genera were calculated using Networkx software (http://networkx.github.io/), with Spearman’s |*r|* > 0.7 and a *p*-value < 0.05 following the previously described method (Ye et al., 2020). Networks visualization was calculated with the interactive platform Gephi (https://gephi.org/). Considering the same nodes existed in the 50 most abundant genera of different treatments, the genera enriched in the OF treatment (vs CF and CK) were grouped together as module 1; the nodes of module 2 and 3 respectively indicated positive and negative correlations to module 1 nodes; the module 4 nodes had no significant correlation with module 1 nodes. Significant differences in each of the soil properties, yield, and Pf/Pi among the three fertilization treatments was determined by ANOVA (at *p* < 0.05).

## Results

### Pf/Pi of CCN Cysts Is Negatively Correlated With Wheat Yield and Soil Total N

Given the hysteresis of fertilization for shifting soil fertility, soil chemistry properties were investigated in the 2^nd^ year trial. After continuously applying the organic fertilizer for two years, the soil total N, total P, available P, available K, and organic matter were 1.19 g kg^-1^, 1.44 g kg^-1^, 115.8 mg kg^-1^, 275.9 mg kg^-1^, and 22.96 g kg^-1^ at wheat jointing stage, respectively (**Table 1**). These soil properties were significantly enhanced over those in treatments with (CF) or without (CK) chemical fertilizer. Therefore, wheat yield was significantly increased by the OF treatment. Interestingly, the index of Pf/Pi of cereal cyst nematodes (its cyst numbers at the harvesting of wheat comparing to that before sowing) was markedly lower in the OF treatment (**Table 1**).

**Table 1:** Soil properties at jointing stage of wheat, wheat yield and Pf/Pi of cereal cyst nematode treated by chemical fertilizer (CF), organic fertilizer (OF) or without fertilizer (CK) treatments in the 2^nd^ year.

Treatment	pH	Organic Matter (g/kg)	Available P (mg/kg)	Total P (g/kg)	Available K (mg/kg)	Total K (g/kg)	Total N (g/kg)	Yield (kg/ha)	Pf/Pi
CK	7.48 ± 0.01^a^	18.34 ± 0.55^b^	34.92 ± 1.46^b^	1.168 ± 0.021^b^	181.8 ± 9.2^b^	6.624 ± 0.182^a^	0.857 ± 0.071^b^	5503 ± 421^c^	3.046 ± 0.779^a^
CF	7.34 ± 0.06^b^	18.62 ± 1.11^b^	44.61 ± 2.09^b^	1.183 ± 0.043^b^	153.9 ± 13.1^b^	6.606 ± 0.362^a^	0.949 ± 0.019^b^	7066 ± 172^b^	1.664 ± 0.293^b^
OF	7.37 ± 0.01^ab^	22.96 ± 1.41^a^	115.8 ± 7.0^a^	1.440 ± 0.045^a^	275.9 ± 37.4^a^	7.026 ± 0.250^a^	1.189 ± 0.061^a^	7857 ± 110^a^	1.160 ± 0.237^c^

Values are mean ± SE. Different letters indicate significant differences from one another (P < 0.05) as identified by one-way ANOVA.

**Figure 1:**
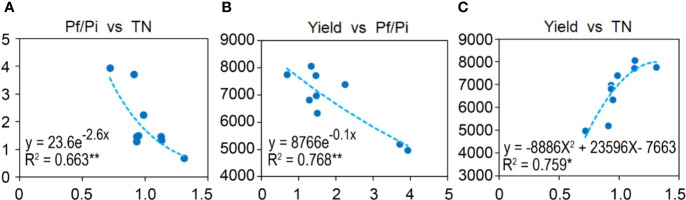
Correlations between **(A)** Pf/Pi and TN, **(B)** Yield and Pf/Pi, or **(C)** Yield and TN. Curvilinear of soil and wheat yield in the 2^nd^ year. * represents the *p* < 0.05, ** represents the *p* < 0.01.

To understand the relationships among Pf/Pi, soil chemical properties, and wheat yield, a heatmap of bivariate Pearson correlations was generated (**Figure S1**). The Pf/Pi had significant curvilinear negative correlation with soil total N (TN) (R^2^ = 0.6631, *p* < 0.01) and yield of wheat (R^2^ = 0.7680, *p* < 0.01) (**Figures 1A, B**). The yield of wheat had a significant curvilinear positive correlation with soil TN (R^2^ = 0.759, *p* < 0.05) (**Figure 1C**).

### General Analyses of the Hi-Seq Sequencing Data

After filtering out any chimeric sequences and mismatches, the number of ITS region-reads obtained from the 126 samples totaled 7,836,371. Based on a 97% similarity threshold, a total of 2631 OTUs were obtained for fungi across all the samples. Rarefaction curves of most samples tended to be flatten (**Figure S2**), suggesting that a reasonable sequencing depth was achieved (although extra rare fungal taxa were likely present in the samples). This robust sampling extent was further supported by the high coverage estimates obtained (**Tables 2** and **S1**).

**Table 2:** Richness and diversity estimation of the ITS sequencing libraries in CK, CF, and OF treatments in the 2^nd^ year trial.

α Diversity		Wheat growth stages						
		Before sowing	Seedling	Wintering	Regreening	Jointing	Heading	Harvest
Sobs	CK	425 ± 12	474 ± 14	541 ± 31	476 ± 12	405 ± 54	537 ± 40	484 ± 24
	CF	451 ± 48	392 ± 88	452 ± 18*	499 ± 47	457 ± 3	473 ± 22	421 ± 38
	OF	423 ± 53	385 ± 36*	414 ± 90	428 ± 23*	469 ± 40	446 ± 57	479 ± 31
Chao1	CK	548 ± 2	644 ± 12	653 ± 9	650 ± 16	547 ± 43	664 ± 68	618 ± 40
	CF	560 ± 66	542 ± 130	578 ± 48	642 ± 58	596 ± 32	652 ± 65	537 ± 39
	OF	538 ± 60	512 ± 54*	575 ± 132	552 ± 56	596 ± 85	589 ± 65	614 ± 41
Shannon	CK	3.66 ± 0.04	3.70 ± 0.16	4.08 ± 0.34	3.72 ± 0.13	2.34 ± 0.51	3.81 ± 0.36	3.94 ± 0.29
	CF	3.51 ± 0.26	2.76 ± 1.20	3.58 ± 0.23	3.91 ± 0.17	3.46 ± 0.24*	3.55 ± 0.26	3.66 ± 0.20
	OF	3.53 ± 0.37	2.84 ± 0.30*	3.29 ± 0.42	3.66 ± 0.05	3.59 ± 0.36*	3.54 ± 0.34	3.89 ± 0.31
Simpson	CK	0.057 ± 0.005	0.051 ± 0.009	0.044 ± 0.016	0.059 ± 0.012	0.351 ± 0.115	0.063 ± 0.028	0.057 ± 0.023
	CF	0.095 ± 0.027	0.248 ± 0.228	0.080 ± 0.019	0.047 ± 0.011	0.087 ± 0.050*	0.082 ± 0.032	0.057 ± 0.010
	OF	0.074 ± 0.059	0.155 ± 0.077	0.092 ± 0.031	0.055 ± 0.005	0.077 ± 0.045*	0.075 ± 0.033	0.057 ± 0.025
Coverage	CK	0.996 ± 0.001	0.996 ± 0.001	0.996 ± 0.001	0.996 ± 0.001	0.996 ± 0.001	0.996 ± 0.001	0.996 ± 0.001
	CF	0.997 ± 0.001	0.996 ± 0.001	0.996 ± 0.001	0.996 ± 0.001	0.996 ± 0.001	0.996 ± 0.001	0.997 ± 0.001
	OF	0.997 ± 0.001	0.996 ± 0.001	0.996 ± 0.001	0.996 ± 0.001	0.996 ± 0.001	0.996 ± 0.001	0.996 ± 0.001

CK, without fertilizer; CF, chemical fertilizer; OF, organic fertilizer. Values are mean ± SE. Asterisks indicate statistically significant differences compare to CK in same column by t-test: *p < 0.05.

### Influence of Organic Fertilizer on α-Diversity and β-Diversity of Fungal Communities

**Figure 2:**
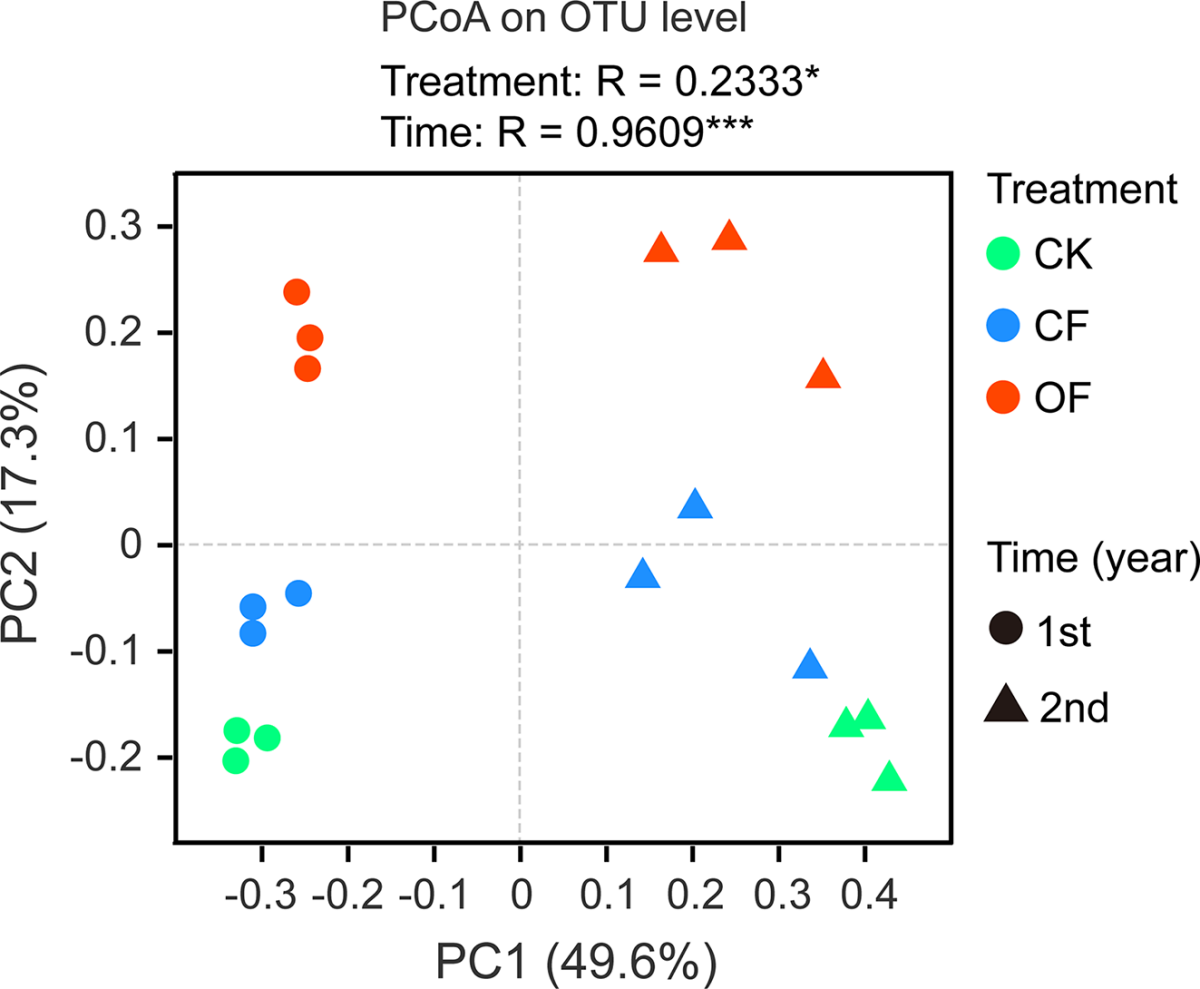
Principal co-ordinate analysis (PCoA) utilizing the bray-curtis distances on operational taxonomic unit (out) level at wheat jointing stage in two years trial. CK, without fertilizer; CF, chemical fertilizer; OF, organic fertilizer. Asterisks indicate statistically significant differences by ANOSIM analysis: *p < 0.05, ***p < 0.001.

The α-diversity of fungal communities in each year trial were compared among OF, CF, and CK treatments at different wheat growth stages (**Tables 2** and **S1**). Significant differences were observed from the seedling through to jointing stages of wheat. At the seedling stage, community richness indices (including Sobs index and Chao1 index) were lower in the OF than CK treatment in both years. After continuous organic fertilizer application, higher fungal community diversity (including Shannon index and Simpson index) was mainly observed under this treatment at the jointing stage of wheat in the 2^nd^ year trial (**Table 2**). Additionally, PCoA was done at the OTU level to infer the similarity of soil fungal communities at wheat’s jointing stage among different treatments (**Figure 2**). The OF samples were obviously separated from that of CF or CK in both years. The ANOSIM analysis revealed a clear separation of treatment (R = 0.2333, *p* < 0.05) and time (R = 0.9609, *p* < 0.001).

### Factors Influencing Shifts in Soil Fungal Communities and CCN Under Organic Fertilizer

**Figure 3:**
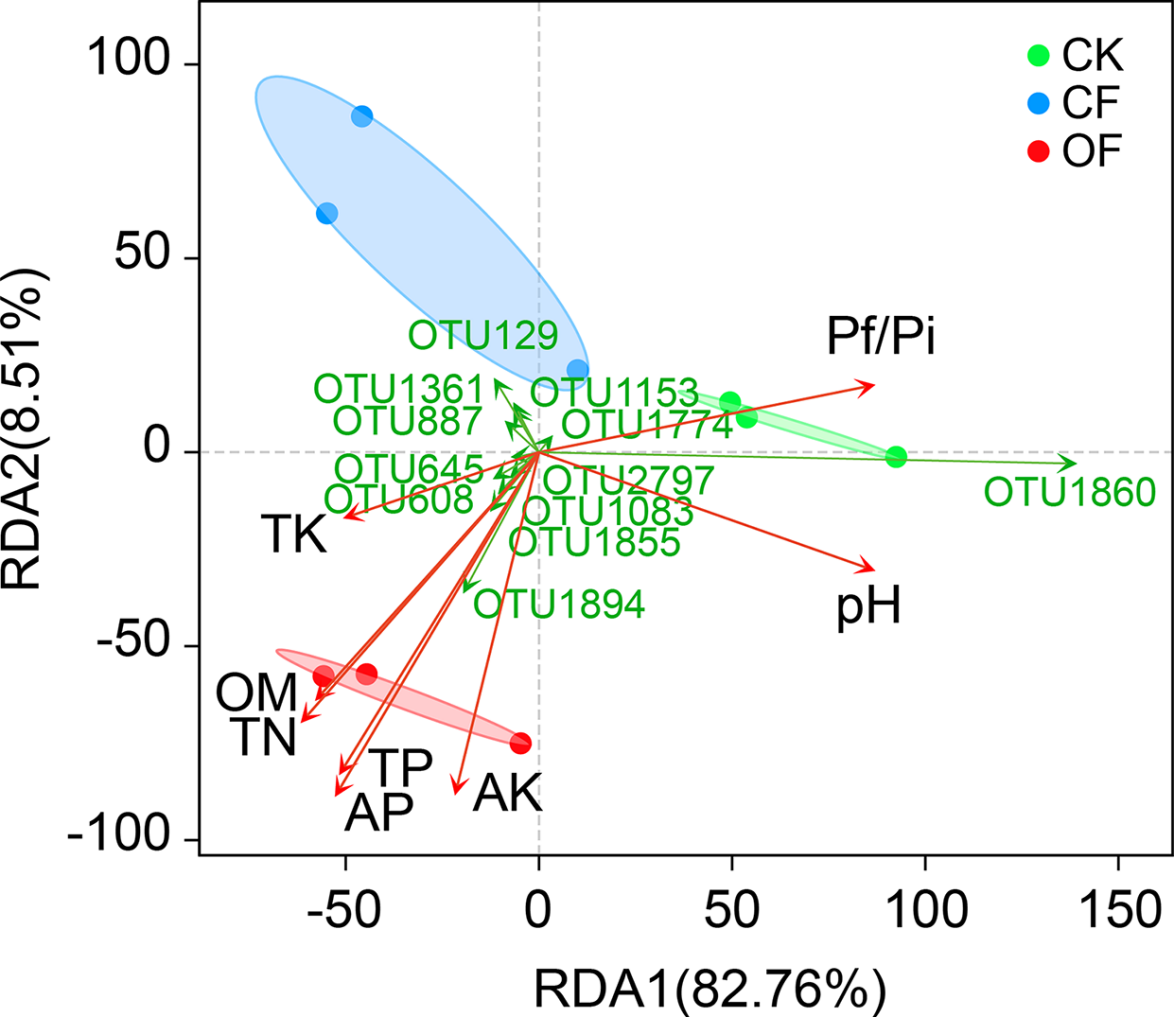
Redundancy analysis (RDA) on operational taxonomic unit (OTU) level, soil properties, and Pf/Pi of cereal cyst nematode at wheat jointing stage in 2^nd^ year trial. CK, without fertilizer; CF, chemical fertilizer; OF, organic fertilizer. Soil properties: TN, total N; TP, total P; TK, total K; AP, available P; AK, available K; OM, organic matter. The OTUs represent the 12 most abundant species.

RDA was used to determine the correlation of soil properties (**Table 1**) with fungal community structures at the wheat jointing stage (**Figure 3**). These soil variables evidently influenced the fungal community, with the first two axes explaining 82.76% and 8.51% of variance in the species’ compositional data, respectively. This revealed that total N, total P, available P, and available K were significantly and positively correlated with fungal community structure in the OF treatment. Particularly, both available P and total P were the two most important contributors (*r^2^* = 0.905 and 0.827, respectively) to variation in the fungal communities (**Table S2**). Interestingly, soil fungal communities treated with OF showed significant negative correlations with Pf/Pi (**Figure 3**) which was consistent with the down-regulation of Pf/Pi of cereal cyst nematode found in the OF treatment. Additionally, of the 12 most abundant OTUs, six were positively related to the OF treatment. This number was more than that of positively related OTUs in CF or CK treatment.

### Negative Correlations Between the Enriched Genera of Fungi and Pf/Pi of CCN in the OF Treatment

**Figure 4:**
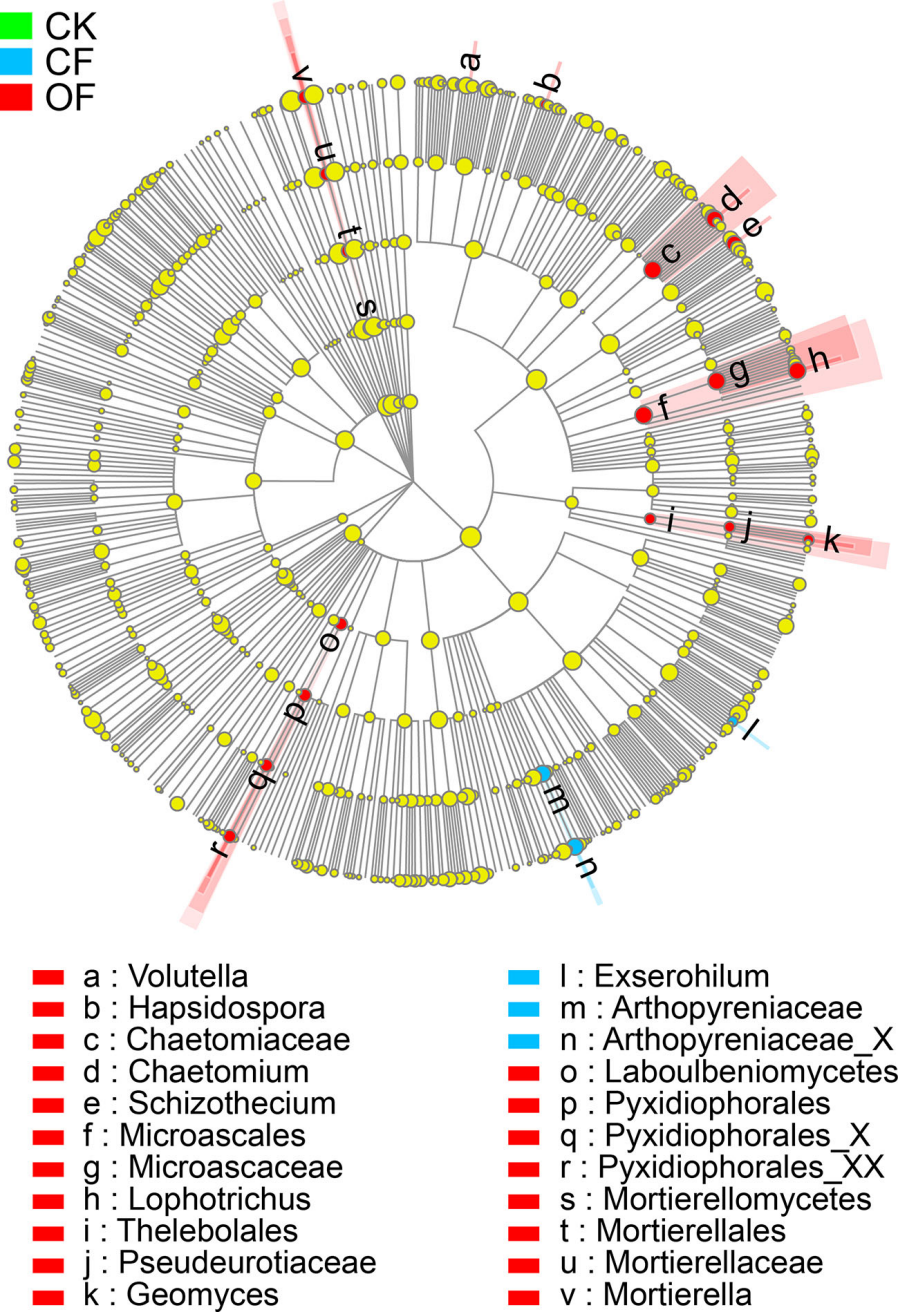
Cladogram showing the phylogenetic distribution of fungal lineages in soils. The taxa of fungi with significantly enriched abundances treated with organic fertilizer (OF), chemical fertilizer (CF), or without fertilizer (CK) are represented by red, blue, or green dots, respectively. The taxa with the absolute LDA scores >2.5 and *p* < 0.05 are shown.

To investigate the distinct taxonomic profiles in OF treatment at the wheat jointing stage in the 2^nd^ year trial, the abundance enriched fungal taxa in each treatment against the others were identified by LDA measurements (**Figures 4** and **S3B**). The numbers of enriched taxa were much higher in the OF treatment than those of either the CF or CK treatments. The significantly enriched taxa numbers in the OF, CF, and CK treatments respectively were 19, 3, and 0. Under the OF, *Volutella* (the genus), *Hapsidospora* (genus), *Chaetomiaceae* (the family and its genus *Chaetomium*), *Schizothecium* (genus), *Microascales* (the order, and its family *Microascaceae* and genus *Lophotrichus*), *Thelebolales* (the order, and its family *Pseudeurotiaceae* and genus *Geomyces*), *Laboulbeniomycetes* (from class to genus), and *Mortierellomycetes* (class to genus) were all enriched. In stark contrast, only *Exserohilum* (genus) and *Arthopyreniaceae* (family to genus) were enriched by the CF treatment.

**Figure 5:**
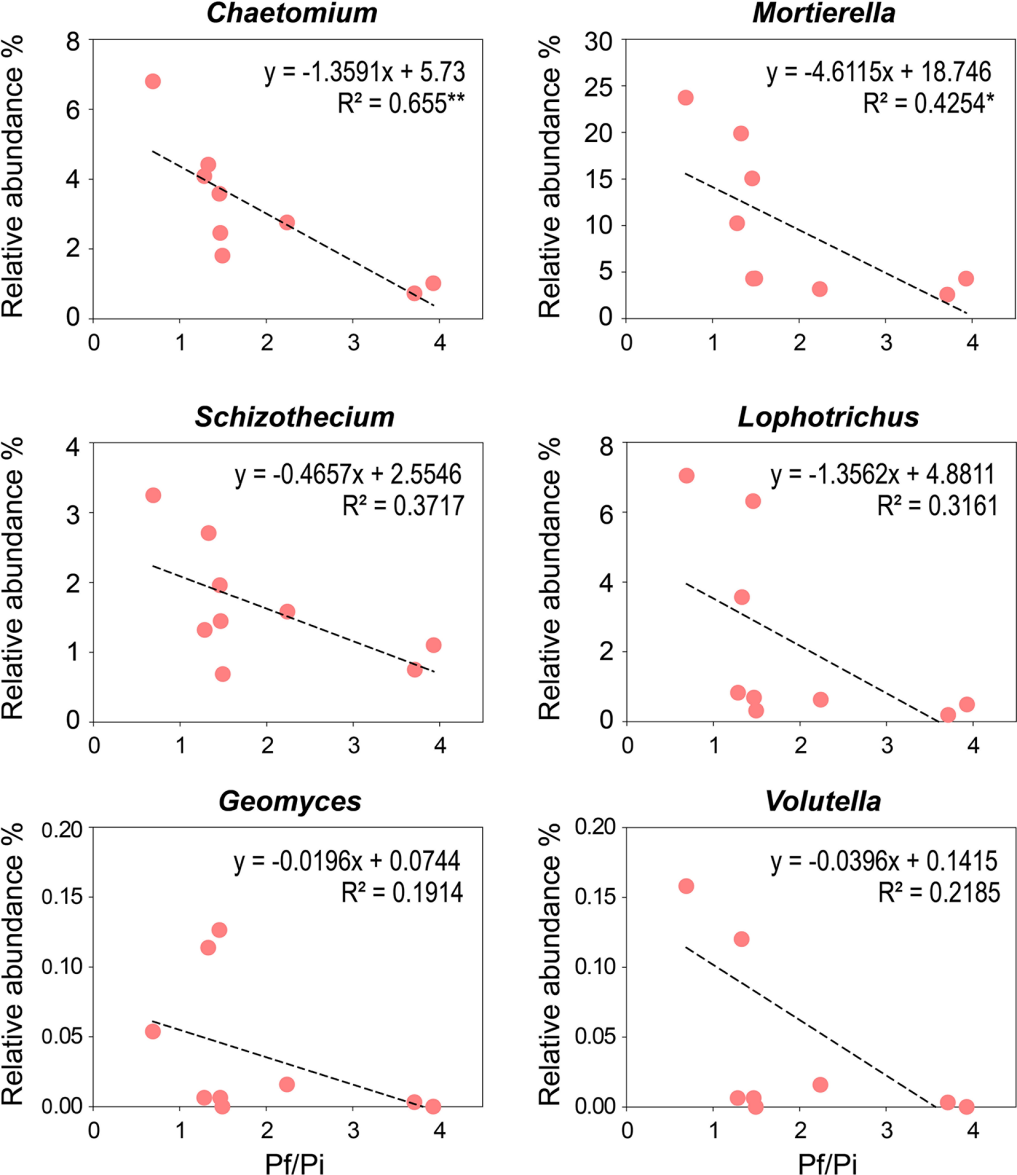
Negative correlations between Pf/Pi of cereal cyst nematode and the enriching genus (*Chaetomium, Mortierella, Schizothecium, Lophotrichus, Geomyces*, and *Volutella*) observed in organic fertilizer treatment at wheat jointing stage in 2^nd^ year trial. * represents the p < 0.05, ** represents the p < 0.01.

To determine the relationships between Pf/Pi of cereal cyst nematode and the relative abundance of enriched genera observed in the OF treatment, linear correlation analyses were conducted (**Figure 5**). The relative abundances of the eight enriched genera, presented in **Table S3**, were negatively correlated with the Pf/Pi of CCN, except those of *Hapsidospora* and *Pyxidiophorales_XX*, which occurred only in the OF treatment at a relative abundance of 0.08% and 0.34%, respectively. Notably, the abundances of genera *Chaetomium* (4.9%) and *Mortierella* (19.6%), showed a significantly negative correlation with the Pf/Pi of CCN. Interestingly, the *Lophotrichus, Mortierella*, and *Geomyces* genera were influenced by organic fertilization greatly, as reflected by their identical enrichment in both trial years (**Figure S3**).

To parse the fungal community datasets by ecological guild, FUNGuild was used, an efficient and widely used method. In comparison with the CK and CF treatments, trophic mode (pathotroph, saprotroph, symbiotroph) and guild (especially that of pathogen) of the eight enriched fungal genera in the OF treatment were analyzed (**Table 3**). Except for the unmatched genus *Pyxidiophorales_XX*, the other unique genus *Hapsidospora* in OF treatment was designated a saprotroph. Additionally, genera *Geomyces*, *Lophotrichus*, and *Schizothecium* also belonged to the saprotroph guild. Mortierellomycota was classified into both saprotroph and symbiotroph. Interestingly, *Chaetomium* and *Volutella* were each a pathotroph. The FUNGuild database predicted *Chaetomium* as an animal pathogen; hence it could inhibit soil animals such as nematodes.

**Table 3:** Functional profile of the enriched genus in organic fertilizer treatment predicted by FUNGuild.

Phylum	Genus	Trophic Mode	Guild	References
*Ascomycota*	*Chaetomium*	Pathotroph/ Saprotroph/ Symbiotroph	Animal Pathogen/ Dung/Plant/Wood Saprotroph/ Endophyte/Epiphyte	Bills et al. (2013) Tedersoo et al. (2014) Massimo et al. (2015) Busby et al. (2016) David et al. (2016)
*Ascomycota*	*Volutella*	Pathotroph	Plant Pathogen	Tedersoo et al. (2014)
*Ascomycota*	*Hapsidospora*	Saprotroph	Undefined Saprotroph	Sterkenburg et al. (2015)
*Ascomycota*	*Geomyces*	Saprotroph	Soil Saprotroph	Minnis and Lindner (2013) Tedersoo et al. (2014)
*Ascomycota*	*Lophotrichus*	Saprotroph	Undefined Saprotroph	Tedersoo et al. (2014)
*Ascomycota*	*Schizothecium*	Saprotroph	Dung Saprotroph	Bills et al. (2013) Tedersoo et al. (2014)
*Mortierellomycota*	*Mortierella*	Symbiotroph/ Saprotroph	Endophyte/Litter/Soil Saprotroph	Cannon and Kirk (2007) Purahong et al. (2016)
*Ascomycota*	*Pyxidiophorales_XX*	–	–	–

‘–’ indicates no matched function in FUNGuild data base.

### More Taxa Correlated With the Enriched Genera in the OF Treatment

**Figure 6:**
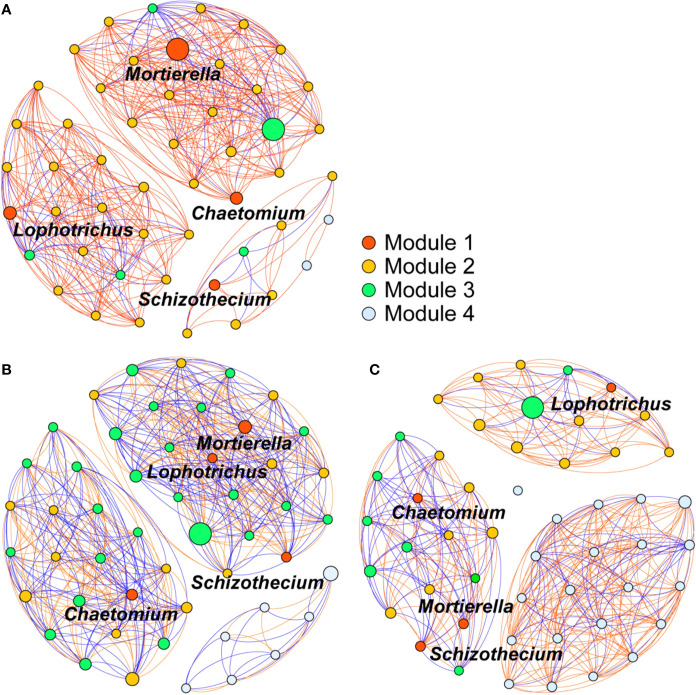
Co-occurrence network of the 50 most abundant soil fungi (genus level) treated with organic fertilizer **(A)**, chemical fertilizer **(B)**, or without fertilizer **(C)**. Module 1 nodes indicate the enriched genus observed in organic fertilizer treatment. Nodes of module 2 and 3 indicate the positive and negative correlations to module 1 nodes, respectively. Module 4 nodes indicate no significant (*p* < 0.05) correlation with module 1. Each edge stands for (Spearman’s |*r|* > 0.7) correlations. Red edges represent positive correlations, blue edges represent negative correlations. The size of each node is relative abundance of the genus.

The 50 most abundant soil fungal genera, constituted an intense community network that differed considerably among the OF, CF, and CK treatments (**Figure 6**). Among the eight fungal genera enriched by organic fertilization, those of *Geomyces*, *Lophotrichus*, *Chaetomium*, and *Mortierella* belonged to the groups of 50 most-abundant genera. To analyze associations between the enriched genera and others, the former were designated as module 1 nodes (red color), for which the nodes of module 2 and 3 respective indicate the positive and negative correlations, while the module 4 nodes had no significant (*p* < 0.05) correlation with module 1. This network analysis revealed that more genera were significantly related to module 1 nodes in the OF treatment than in the CF and CK treatments (44, 39, and 25 genera, respectively). Under the OF treatment, the number of positive and negative genera were 39 and 5, respectively. Meanwhile, the percentage of positive relation among different nodes in OF was 81%, much higher than CF’s 52% or CK’s 65%. The higher relative abundance—as conveyed by the size of each node—of module 1 nodes and its 39 significant positively correlated fungal genera constituted a more intense community network in the soils treated with the organic fertilizer.

## Discussion

### Organic Fertilization Reduces Pf/Pi of CCN to Enhance Wheat Yield

Applying organic fertilizers (OF) is able to improve soil fertility and wheat yield, but it can also effectively reduce the damage to crops from cereal cyst nematode [CCN] (Diacono and Montemurro, 2010; Hu et al., 2014). A similar phenomenon was found in our present study. Compared with CF, the OF treatment significantly enhanced soil organic matter and improve other soil properties, while decreasing the Pf/Pi of CCN (**Table 1**). Some studies have pointed out that a few different elemental fertilizers can exhibit various effects on CCN populations. For instance, nitrogen and phosphate fertilizers, especially urea or calcium superphosphate, are effective at suppressing the abundance of CCN or plant parasitic nematodes (Al-Hazimi and Dacbah, 2014; Zhao et al., 2014; Song et al., 2015). Potassium sulfate promotes whereas ammonium sulfate alleviates the damage to plants caused by CCN (Yang et al., 2008; Ikoyi et al., 2020), thus implicating potassium a plausible key factor promoting CCN’s abundance in fields (Yang et al., 2008). In our study, the integrated shifts of soil properties in the OF treatment might have reduced the Pf/Pi of CCN, with soil nitrogen having the strongest effect (significant negative correlation with Pf/Pi) on suppressing CCN reproduction. Ultimately, the suppressed abundance of plant parasitic nematodes will likely ensue with the addition of organic materials over the long-term (i.e., more than 10 years) in such fertilization experiments (Li et al., 2010). In addition, in wheat fields treated with a gradient of nitrogen applications, plant parasitic nematodes attained their highest numbers at 100 kg N ha^-1^ application, but decreased at 300 kg N ha^-1^ application (Song et al., 2015). Hence, the quantity of application of fertilizers may also affect the community structure of soil nematodes, as well as the abundance of those that are plant parasitic nematodes.

The type of organic materials used in fields may also influence differential suppression of plant parasitic nematodes in soils (Li et al., 2010; Roth et al., 2015). For example, sugarcane bagasse and sugarcane refinery sludge were used as organic inputs in a banana field, where their application suppressed the relative abundance of plant parasitic nematodes more so than the application of plant residues (Tabarantab et al., 2011), and the latter’s effects on plant parasites also differed from those of animal manure. Recently, Li et al. (2018) found that the relative abundance of plant parasites was highest in a polar leaf addition, followed by maize straw, yet it was lowest in cow manure treatments. Therefore, it seems that a more suitable soil environment for plant parasitic nematodes is created by plant residues application. Nevertheless, different effects from various kinds of animal manures upon CCN, such as chicken manure versus cow manure, have yet to be characterized.

### Organic Fertilization Promotes the Enrichment of Fungal Communities Negatively Related to CCN

Fertilization of soil, through organic or chemical fertilizers, may affect rhizosphere fungal communities by changing their food or energy sources in terms of the quality and quantity of root exudates (Kerry, 2000; Nuland et al., 2016; Zhang et al., 2017). In this study, the induced changes to soil fungal communities mainly occurred from the seedling through jointing stage of wheat (**Tables 2** and **S1**). Higher fungal community diversity was not observed until the jointing stage in the 2^nd^ year trial, a result that may be explained by the hysteresis of continuous organic fertilization. Similarly, fungal community structures under the OF treatment were distinct and unlike the other treatments in the 2^nd^ year trial (**Figure 2**), indicating a significant influence on the rhizosphere ecosystem from continuous organic fertilization over two successive years. Interestingly, it precisely the wheat jointing stage that has the most suitable soil temperature for cereal cyst nematodes’ egg hatching and juvenile penetration into wheat roots, thus presenting the highest risk of parasitism to wheat roots (Yuan et al., 2014; Song et al., 2015). Therefore, the jointing stage is not only a critical period for wheat yield but also a critical period for the interaction between CCN parasitism and soil fungal communities.

Organic fertilization offers a valuable way to restore soil biodiversity that is essential for developing sustainable agriculture, which has been severely undermined by intensive farming based on the widespread use of synthetic pesticides and chemical fertilizers (Glen-Karolczyk et al., 2018). Organic inputs, as a critical source of organic matter, can enhance the abundance of plant beneficial fungi that have been inhibited from the excessive use of chemical fertilizer during intensive agriculture (Ai et al., 2015), In our study, applying organic fertilizer significantly enhanced the α-diversity (Shannon and Simpson) and resulted in distinct community composition (PCoA) of rhizosphere fungal communities (**Table 2** and **Figure 2**), and promoted positive correlations between fungal community structures and key soil chemical properties (total N, total P, available P, available K) at wheat jointing stage (**Figure 3**). Furthermore, much more enriched fungus taxa were observed in the wheat rhizosphere (**Figure 4**), suggesting that organic inputs contributed to a suitable growth substrate or environment for fungi (Bernard et al., 2014). This finding is consistent with the view that soil fungal communities could be altered through an enhancement of soil chemical properties by fertilization practices (Ai et al., 2015; Yao et al., 2018).

Soil fungal community composition, diversity, and functioning have proven links to the occurrence of plant soil-borne disease (Shen et al., 2019). A large abundance of microorganisms will accumulate on pathogen-suppressive soil, and they are negatively correlated with pathogens’ abundance (Raaijmakers and Mazzola, 2016; Hamid et al., 2017). Similarly, corresponding to the down regulation the Pf/Pi of CCN, the soil treated with organic fertilizer harbored fungal community structures which showed significantly negative correlations with the Pf/Pi of the nematode (**Figure 3**). Among the eight fungi genera enriched by organic fertilization, *Chaetomium* and *Mortierella* had negative correlations with the Pf/Pi of CCN (**Figure 5**). Further, a more intense community network of rhizosphere fungi formed under the OF treatment (**Figure 6**), suggesting these fungi may have functions for suppressing CCN. Generally, natural disease suppression does not rely on a single taxon but rather the high abundance of several microbial taxa, or special functional groups of microorganisms, corresponding to disease suppression (Sanguin et al., 2009; Mendes et al., 2011; Chapelle et al., 2016). Yet rare populations may also exert a disproportionately large effect on a community’s functional stability if they provide a common good, such as secreted extracellular enzymes or essential growth factors that other members are incapable of synthesizing (Konopka et al., 2015). Accordingly, the unique existence of genera *Hapsidospora* and *Pyxidiophorales_XX* in the OF treatment should not be overlooked, despite their very low relative abundance of 0.08% and 0.34%, respectively.

### The Assembled Soil Fungi Are Candidate Biocontrol Agents for CCN

Nematophagous fungi have been extensively investigated as biological control agents of nematodes since the 20th century (Barron, 1977). Two of the enriched soil fungi genera in the OF treatment, *Mortierella* and *Chaetomium*, are known to have the ability to suppress many pathogens (Edgington et al., 2014; Melo et al., 2014; DiLegge et al., 2019). Overall, their functional mechanisms are related to antibiotics’ synthesis and internal parasitism. For example, *M. alpina* can synthesize alkaloid antibiotics and possess marked biocontrol activity against some human pathogens (Melo et al., 2014). These compounds have been isolated from the endophytes *Neotyphodium* spp. of grasses and then used to enhance the protection of plants against worms and phytopathogens (Schardl et al., 2004; Zhang et al., 2012). Furthermore, both *M. alpina* and *M. signyensis* are apt at killing insect pests, such as the wax moth (*Galleria mellonella* L.) and housefly (*Musca domestica* L.), by inoculation or injection (Edgington et al., 2014). As to another ecological function of *Mortierella*, the species *M. globalpina* was confirmed as pathogenic against root-knot nematodes by trapping them, then penetrating and digesting the nematodes’ cellular contents (DiLegge et al., 2019).

Besides *Mortierella*, species of the genus *Chaetomium* have been also investigated as potential biological control agents against soil-borne diseases (Tagawa et al., 2010). The *Chaetomium* members are important candidate nematode-controlling fungi, distinguished by their biosynthesis of chaetoglobosins, which are effective preventive agents against plant parasitic nematodes (Nitao et al., 2002; Hu et al., 2013). *Chaetomium* is a widespread genus, with 95 known species from around the world (Kirk et al., 2008), which can produce a diverse array of secondary metabolites: not only chaetoglobosins, but also xanthones, anthraquinones, terpenoids, depsidones, and steroids having anticancer, antioxidant, antimicrobial, and cytotoxic properties, among others (Zhang et al., 2012). *C. globosum* is reportedly an effective biocontrol agent for several plant pathogenic microorganisms (Qin et al., 2009; Shanthiyaa et al., 2013), and its metabolites have been tested and confirmed for nematicidal activities against *Meloidogyne incognita* and *H. glycines* (Nitao et al., 2002; Hu et al., 2013). Chaetoglobosin A is the major component of the chaetoglobosins synthesized by *C. globosum* (Qin et al., 2009; Kawahara et al., 2013). A temporary inhibition of mobility was observed when chaetoglobosin A was tested against *Caenorhabditis elegans* and *H. filipjevi* (Ashrafi et al., 2017), and it has toxic, lethal effects against *M. incognita* (Hu et al., 2013).

Along with the *Mortierella* and *Chaetomium*, the *Geomyces* and *Lophotrichus* are noteworthy; together, these were the four most abundant genera enriched by OF (**Table S3**). In our network analysis, the hubs indicated the most important nodes, which may be interpreted as being the key taxa inside a connected community (Gu et al., 2020). We found these four genera negatively related to the Pf/Pi of cereal cyst nematode, and they were surrounded by much more closely associated genera, suggesting that organic fertilization assembles the fungal communities that suppress CCN in the rhizosphere of wheat plants.

## Conclusion

Our study provided an integrative view of the relationship between cereal cyst nematode (CCN) and soil fungal communities as shaped by organic fertilizer applications in wheat field of the Kaifeng district, in China’s Henan province. The organic fertilizer treatment enhanced soil total N, as well as soil organic matter, total P, available P, and available K at the jointing stage, when both egg hatching and J2 penetrating into root of wheat plant by CCN is greatest. We found that soil total N was positively correlated with wheat yield and soil fungal community structures, but negatively correlated with the Pf/Pi of cereal cyst nematode. Our results implied that integrated shifts of soil properties are a key factor contributing to the reduced index of Pf/Pi of CCN under OF; in particular, the negative effect of soil nitrogen was the most powerful at suppressing CCN. Further, organic fertilization improved α-diversity and β-diversity of rhizosphere fungal communities, and enriched several fungal genera, namely *Mortierella*, *Chaetomium*, *Lophotrichus*, *Schizothecium*, *Volutella*, and *Geomyces*, whose abundances were negatively related to the Pf/Pi of CCN. Furthermore, an intense fungal community network, characterized by much more closely related fungi surrounding the nematophagous fungal genus *Mortierella* and *Chaetomium*, suggested that CCN-suppressing fungal community structures were stimulated by organic fertilization. Taken together, organic fertilization enhances soil fertility and assembled fungal communities capable of suppressing cereal cyst nematode reproduction in the rhizosphere, resulting in wheat yield’s increase. Our results help to understand deeply the relationships among fertilization, soil-borne disease, and soil fungal communities.

## Data Availability Statement

The datasets generated and analyzed during the current study are available in the NCBI Sequence Read Archive (SRA) repository under the BioProject number PRJNA639921.

## Author Contributions

WQ, QL, and HJ designed the experiments. WQ and HS performed most of the experiments. KJ and LY participated in part of the soil analysis. WQ wrote the manuscript. HS, QL, and HJ revised the manuscript. All authors discussed the results and commented on the manuscript. HJ provided funding for this work as the corresponding author.

## Funding

This study was supported by the National Key Research and Development Program of China (Grant No. 2017YFD0200601), and a grant from the National Natural Science Foundation of China (NSFC, Grant No. 31871940).

## Conflict of Interest

The authors declare that the research was conducted in the absence of any commercial or financial relationships that could be construed as a potential conflict of interest.

## Supplementary Material

Table S1 Richness and diversity estimation of the ITS sequencing libraries in CK, CF, and OF treatments in the 1^st^ year trial. CK: without fertilizer; CF: chemical fertilizer; OF: organic fertilizer. Values are mean ± SE. Asterisks indicate statistically significant differences compare to CK in same column by t-test: * *p* < 0.05.

Table S2 Explanation of environmental factors affecting fungal community structure at wheat jointing stage in 2^nd^ year trial. OM, organic matter; AP, available P; TP, total P; AK, available K; TK, total K; TN, total N. Pf/Pi, index of cereal cyst nematode reproduction.

Table S3 Relative abundance of the enriching genus observed in organic fertilizer treatment at wheat jointing stage in 2^nd^ year trial. CK: without fertilizer; CF: chemical fertilizer; OF: organic fertilizer. ‘n’ indicates no abundance.

Figure S1 Pearson relationship among soil properties, wheat yield and Pf/Pi of cereal cyst nematode. * represents the *p* < 0.05. TN: total N; TK: total K; TP: total P; AK: available K; AP: available P; OM: organic matter.

Figure S2 The rarefaction analysis of all samples within the Shannon index (A) and Sobs index (B).

Figure S3 A linear discriminant analysis effect size (LEfSe) method identifies the significant different abundant taxa of fungi in the soil of organic fertilizer (OF), chemical fertilizer (CF), or without fertilizer (CK) treatments at wheat jointing stage. The taxa with the absolute LDA scores > 2.5 and *p* < 0.05 are shown. Red letters indicate the same taxa enriched in two-year trials.
