# Supplementary material for: Organic Fertilization Assembles Fungal Communities of Wheat Rhizosphere Soil and Suppresses the Population Growth of Heterodera avenae in the Field
Source: Front Plant Sci. 2020 Aug 7;11:1225. doi: 10.3389/fpls.2020.01225 (PMC7429443; doi:10.3389/fpls.2020.01225)
Supplement: Table S1 — Richness and diversity estimation of the ITS sequencing libraries in CK, CF, and OF treatments in the 1st year trial. CK: without fertilizer; CF: chemical fertilizer; OF: organic fertilizer. Values are mean ± SE. Asterisks indicate statistically significant differences compare to CK in same column by t-test: * p < 0.05. [file DataSheet_1.pdf]

Supplementary Material

Organic Fertilization Assembles Fungal Communities of Wheat Rhizosphere Soil and suppresses the population growth of *Heterodera avenae* in the Field

Wei Qiu[1], Huiqing Su[1], Lingyun Yan, Kaiyan Ji, Qian Liu, Heng Jian[*]

Department of Plant Pathology and MOA Key Laboratory of Pest Monitoring and Green Management, China Agricultural University, Beijing, China

TABLE S1 | Richness and diversity estimation of the ITS sequencing libraries in CK, CF, and OF in the 1st year trial.

**α Diversity**		**Wheat growth stages**						
		**Before sowing**	**Seedling**	**Wintering**	**Regreening**	**Jointing**	**Heading**	**Harvest**
Sobs	CK	349±41	383±6	193±20	387±9	333±2	411±20	356±7
	CF	361±31	369±3**	226±12	302±13*	392±16*	361±13	412±19
	OF	384±30	314±11*	191±31	301±6*	358±20	320±24	355±26
Chao1	CK	466±40	549±12	277±39	501±6	424±5	550±44	532±15
	CF	467±31	486±10*	313±18	410±24	524±16*	501±35	548±37
	OF	504±27	430±20**	273±62	437±7*	474±33	428±30	486±31
Shannon	CK	2.29±0.45	3.63±0.03	1.25±0.28	3.73±0.05	3.68±0.05	3.46±0.10	3.19±0.13
	CF	2.64±0.66	3.35±0.09	2.13±0.13	3.10±0.19	3.52±0.19	3.36±0.06	3.49±0.18
	OF	2.59±0.53	2.87±0.39	1.87±0.48	3.35±0.09*	3.58±0.14	2.82±0.30	3.19±0.15
Simpson	CK	0.295±0.096	0.060±0.002	0.618±0.090	0.047±0.003	0.045±0.004	0.076±0.002	0.110±0.022
	CF	0.265±0.163	0.092±0.015	0.260±0.037*	0.106±0.026	0.066±0.016	0.087±0.013	0.085±0.016
	OF	0.274±0.123	0.202±0.075	0.396±0.134	0.066±0.009	0.054±0.008	0.192±0.066	0.110±0.023
Coverage	CK	0.997±0.001	0.996±0.001	0.998±0.001	0.997±0.001	0.997±0.001	0.996±0.001	0.996±0.001
	CF	0.997±0.001	0.997±0.001	0.998±0.001	0.997±0.001	0.996±0.001	0.996±0.001	0.996±0.001
	OF	0.997±0.001	0.997±0.001	0.998±0.001	0.997±0.001	0.996±0.001	0.997±0.001	0.997±0.001

CK: without fertilizer; CF: chemical fertilizer; OF: organic fertilizer. Asterisks indicate statistically significant differences compare to each diversity index in same column by t-test: * $p < 0.05$.

TABLE S2 | Explanation of environmental factors affecting fungal community structure at wheat jointing stage in 2nd year trial.

	**RDA1**	**RDA2**	**r^2**	**p_values**
pH	0.94	-0.35	0.755	0.020
OM	-0.68	-0.73	0.621	0.054
AP	-0.51	-0.86	0.905	0.010
TP	-0.52	-0.85	0.827	0.008
AK	-0.22	-0.97	0.744	0.014
TK	-0.97	-0.26	0.228	0.431
TN	-0.66	-0.75	0.734	0.010
Pf/Pi	0.98	0.20	0.678	0.045

OM, organic matter; AP, available P; TP, total P; AK, available K; TK, total K; TN, total N. Pf/Pi, index of cereal cyst nematode reproduction.

TABLE S3 | Relative abundance of the enriching genus observed in organic fertilizer treatment at wheat jointing stage in 2nd year trial.

**Genus**	**Relative abundance %**								
	**CK1**	**CK2**	**CK3**	**CF1**	**CF2**	**CF3**	**OF1**	**OF2**	**OF3**
Hapsidospora	n	n	n	n	n	n	0.006	0.104	0.139
Pyxidiophorales_XX	n	n	n	n	n	n	0.038	0.199	0.771
Volutella	0.003	n	n	0.006	0.016	0.006	0.120	0.158	0.265
Geomyces	0.003	n	n	0.006	0.016	0.006	0.114	0.054	0.126
Mortierella	2.570	4.308	4.286	10.231	3.164	4.289	19.881	23.718	15.054
Lophotrichus	0.190	0.313	0.490	0.825	0.626	0.686	3.562	7.042	6.312
Schizothecium	0.749	0.686	1.100	1.318	1.580	1.444	2.706	3.246	1.960
Chaetomium	0.727	1.805	1.015	4.087	2.756	2.456	4.412	6.799	3.578

CK: without fertilizer; CF: chemical fertilizer; OF: organic fertilizer. ‘n’ indicates no abundance.

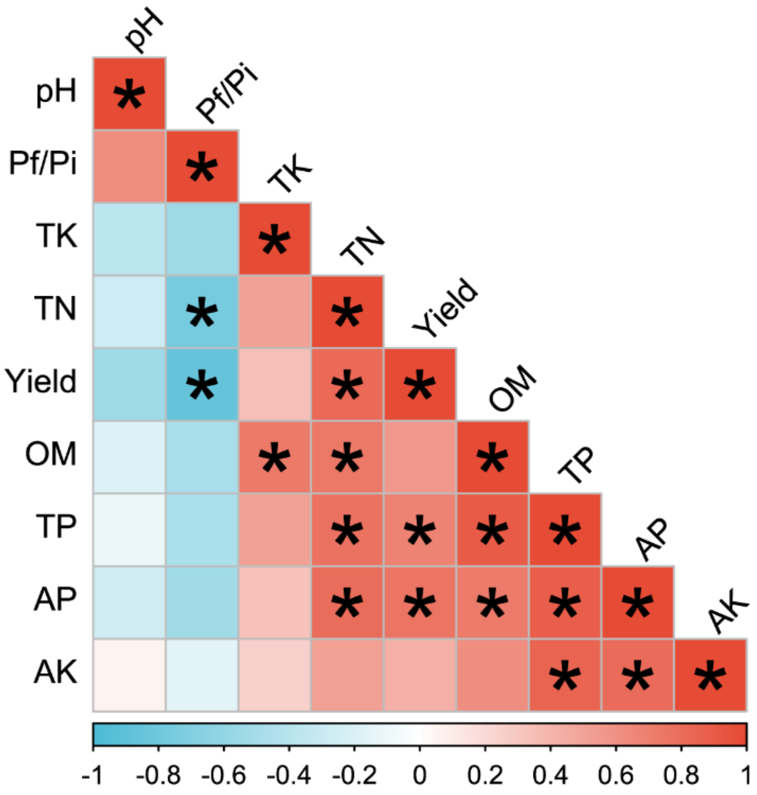

FIGURE S1 | Pearson relationship among soil properties, wheat yield and Pf/Pi of cereal cyst nematode. * represents the $p < 0.05$. TN: total N; TK: total K; TP: total P; AK: available K; AP: available P; OM: organic matter.

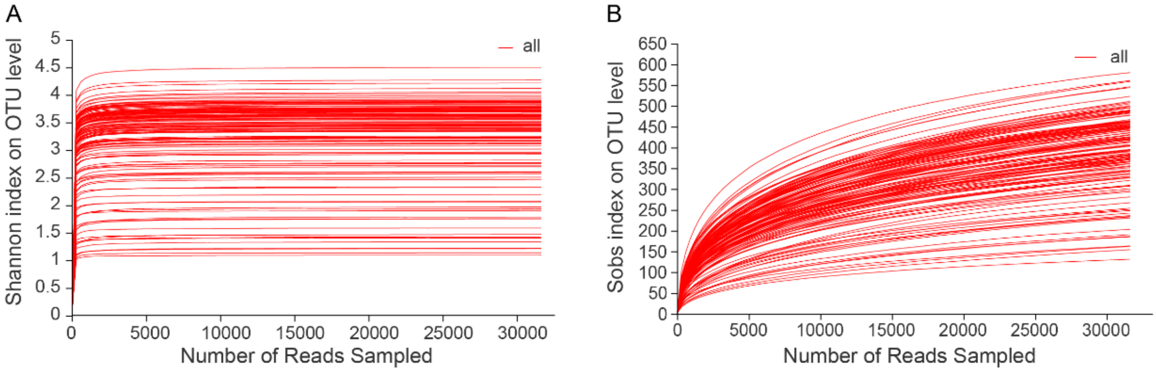

FIGURE S2 | The rarefaction analysis of all samples within the Shannon index (A) and Sobs index (B).

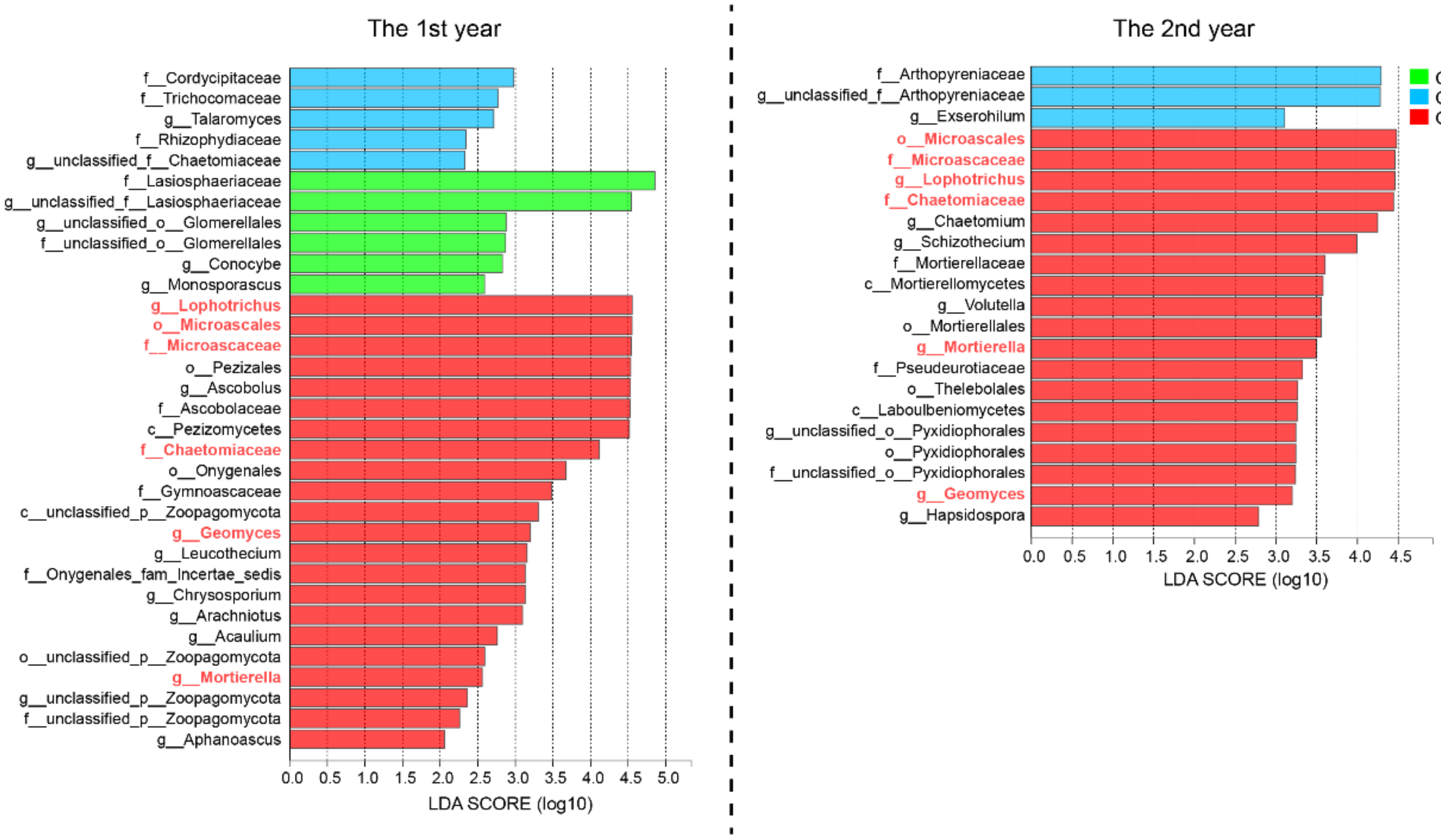

FIGURE S3 | A linear discriminant analysis effect size (LEfSe) method identifies the significant different abundant taxa of fungi in the soil of organic fertilizer (OF), chemical fertilizer (CF), or without fertilizer (CK) treatments at wheat jointing stage. The taxa with the absolute LDA scores > 2.5 and $p < 0.05$ are shown. Red letters indicate the same taxa enriched in two-year trials.
